# Persistent Multiple Colonic Ulcers and Repeated Acute Bloody Diarrhea in an Elderly Patient with Transthyretin Amyloidosis: A Case Report

**DOI:** 10.7759/cureus.68167

**Published:** 2024-08-30

**Authors:** Kenji Yorita, Atsuki Maeda, Kunihisa Uchita, Asuto Nagai, Nobuyuki Tanida

**Affiliations:** 1 Diagnostic Pathology, Japanese Red Cross Kochi Hospital, Kochi, JPN; 2 Internal Medicine, Japanese Red Cross Kochi Hospital, Kochi, JPN; 3 Surgery, Japanese Red Cross Kochi Hospital, Kochi, JPN

**Keywords:** attr amyloidosis, pathology, gallbladder, stomach, erosion, ulcer, colon, transthyretin, amyloidosis

## Abstract

The clinical gastrointestinal manifestations of transthyretin amyloidosis (ATTR amyloidosis) are usually non-specific, and colonic bleeding or ulcers are unusual. Here, we report the case of an elderly Japanese man with ATTR amyloidosis who presented with rare gastrointestinal symptoms. An 84-year-old Japanese man was referred to our hospital because of anemia and positive fecal occult blood test results. Endoscopic examination revealed multiple small colonic erosions and/or ulcers; however, the cause remained unknown. The patient repeatedly visited our hospital for one year because of acute bloody diarrhea, anemia, and multiple persistent colonic ulcers. His symptoms improved after symptomatic treatment. The most recent gastric biopsy revealed ATTR amyloidosis, which was retrospectively detected in colon, stomach, and gallbladder specimens obtained six months, six years, and 12 years earlier, respectively. Therefore, ATTR amyloidosis should be considered in the differential diagnosis of colon-related anemia.

## Introduction

Tissue deposition of protein fibrils causes systemic amyloidosis. Light-chain amyloidosis is the most common type of systemic amyloidosis and transthyretin amyloidosis (ATTR amyloidosis) is increasingly being diagnosed [1]. ATTR amyloidosis is a progressive condition characterized by the extracellular deposition of amyloid fibrils composed of the transthyretin (TTR) protein in various organs and tissues, leading to their dysfunction [2]. The disease manifests in two primary forms: hereditary ATTR amyloidosis (ATTRv), which is autosomal dominant and typically presents earlier in life with a range of symptoms, including polyneuropathy, cardiomyopathy, and other systemic symptoms, and wild-type ATTR amyloidosis (ATTRwt), which typically occurs sporadically in elderly individuals and is frequently associated with cardiomyopathy [3]. Differentiating between ATTRv and ATTRwt amyloidosis is important because of the distinct pathophysiological mechanisms, clinical presentations, and therapeutic approaches associated with each form [4,5]. Recent advancements in therapeutic options, including TTR stabilizers and gene-silencing treatments, have improved the management of this condition [2]. Both forms of ATTR amyloidosis can be treated with TTR stabilizers, such as tafamidis. Thus, clinicopathological identification of ATTR amyloidosis is necessary. The clinical GI manifestations of ATTR amyloidosis can be non-specific, and colonic bleeding or ulcers are unusual [6-9]. Here, we report the case of an elderly Japanese man with ATTR amyloidosis who presented with rare GI symptoms.

## Case presentation

An 84-year-old Japanese man was referred to our hospital because of anemia and positive fecal occult blood test results. He took pravastatin sodium and nizatidine for hyperlipidemia and reflux esophagitis, respectively, but not antiplatelet medications, corticosteroids, or nonsteroidal anti-inflammatory drugs. He had a history of liver cirrhosis due to alcohol consumption, resection of a subcutaneous lipoma of the right shoulder 15 years previously, cholecystectomy for chronic calculous cholecystitis 12 years previously, endoscopic submucosal dissection (ESD) for early gastric cancer (well-differentiated tubular adenocarcinoma/high-grade gastric dysplasia) six years previously, and hemorrhoid surgery six months before presentation. The patient underwent an annual upper gastrointestinal endoscopy after ESD with no recurrence or ulcers. The family history was unremarkable.

Upon admission, vital signs and physical examination revealed no abnormalities. Complete blood and serological tests showed normocytic anemia, elevated white blood cell count and C-reactive protein levels, abnormal renal function, and elevated coagulation time (Table 1).

**Table 1:** Laboratory findings on hospitalization WBC: white blood cells; RBC: red blood cells; MCV: mean corpuscular volume; MCH: mean corpuscular hemoglobin; MCHC: mean corpuscular hemoglobin concentration; AST: aspartate aminotransferase; ALT: alanine aminotransferase; LDH: lactate dehydrogenase; ALP: alkaline phosphatase; BUN: blood urea nitrogen; eGFR: estimated glomerular filtration rate; CRP: C-reactive protein; PT: prothrombin time; APTT: activated partial thromboplastin time; FDP: fibrin degenerated products

	Laboratory parameters	Patient values	Reference range
Complete blood count	WBC	16,210 /µL	3500–8000
	RBC	342×10^4 ^/µL	410–550
	Hemoglobin	9.0 g/dL	13.4–17.4
	MCV	85.4 fL	81.0–100.0
	MCH	26.3 pg	29.2–35.2
	MCHC	30.8 %	30.9–36.1
	Platelet	39.7×10^4 ^/µL	12.3–33.1
Serum chemistry	AST	18 IU/L	10–32
	ALT	15 IU/L	5–27
	LDH	166 IU/L	106–211
	ALP	100 IU/L	38–113
	BUN	19.0 mg/dL	8.0–20.0
	Creatinine	1.2 mg/dL	0.36–1.06
	eGFR	43 ml/min/1.7	
	Na	139 mEq/L	135–150
	Cl	106 mEq/L	98–108
	K	4.2 mEq/L	3.5–5.0
	CRP	7.27 mg/dL	<0.16
Blood coagulation test	PT	14.0 s	9–13
	APTT	27.7 s	20–35
	Fibrinogen	503 mg/dL	170–410
	FDP	8.4 µg/mL	<5
	D-dimer	5.8 µg/mL	<1
Urinalysis	pH	6.5	5.0–8.0
	Specific gravity	1.010	1.001–1.035
	Occult blood	Negative	Negative
	Protein	Negative	Negative
	Bilirubin	Negative	Negative
	Keton	Negative	Negative
	Urobilinogen	Negative	Negative
	Leukocyte esterase reaction	Negative	Negative

**Figure 1:**
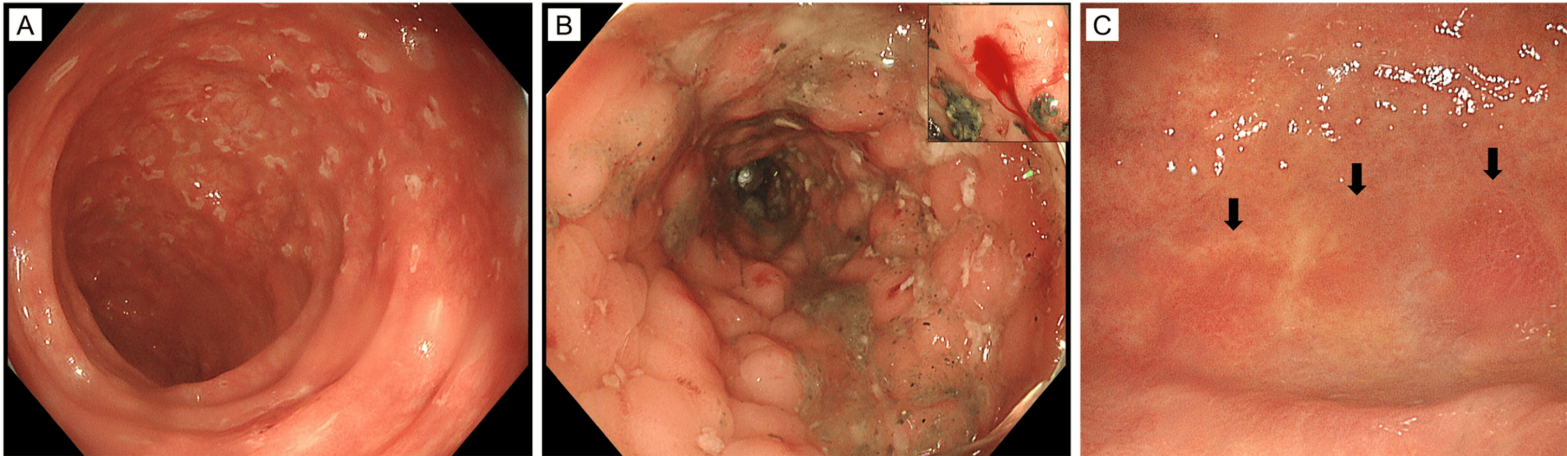
Colonoscopic and gastroscopic images of the elderly patient with ATTR amyloidosis In the absence of active bleeding (A, transverse colon), multiple spotty colonic erosions and/or shallow ulcers were observed in the colon. During active colonic bleeding (B, sigmoid colon), markedly edematous colonic mucosa with multiple erosions, ulcers, or bleeding sites (inset) were observed. Esophagogastroduodenoscopy revealed brownish mucosal areas of the gastric fornix (C, arrows), where ATTR amyloidosis was detected. ATTR: transthyretin amyloidosis

Esophagogastroduodenoscopy confirmed a short Barrett’s esophagus, atrophic gastritis, and no bleeding sites. A colonoscopy revealed multiple small ulcers and/or erosions of < 5 mm from the cecum to the sigmoid colon (Figure 1A). Colonic diverticulosis and absence of bleeding from the diverticula were observed. No pathogenic microorganisms were found in the stool cultures. Colonic biopsies of five lesions showed ulcers but could not reveal the cause. Subsequently, clinical follow-up was performed.

After the first examination, the patient visited our hospital over one year because of acute bloody diarrhea twice and colitis due to enteropathogenic *Escherichia coli* once. The clinicopathological timeline and medical history are presented in Table 2.

**Table 2:** Clinicopathological timeline including medical histories NA: not available; FOB: fecal occult blood; ABD: acute bloody diarrhea; A: ascending colon; S: sigmoid colon; C: cecum; R: rectum; D: descending colon; ESD: endoscopic submucosal dissection; Tis: carcinoma in situ; ATTR: transthyretin amyloidosis; EMR: endoscopic mucosal resection; SS: subserosa; SM: submucosa; M: mucosa

Age (years)	70	73	79	83	84	84	85	85	85	85	
Symptom/clinical finding (hemoglobin)	None (NA)	Epigastric pain (NA)	None (12.7 g/dL)	None (NA)	FOB (9.0 g/dL)	ABD (7.9 g/dL)	Diarrhea (10.8 g/dL)	None (NA)	ABD (7.5 g/dL)		
Site of biopsy or surgery, ESD,	Subcutaneous tissue of the shoulder	Gallbladder	Stomach	Stomach	Colon	Colorectum	Colorectum	Colon	Colorectum		Stomach
Clinical findings or diagnosis	Lipoma, suspected	Cholecystolithiasis	Early gastric cancer, suspected	Red gastric mucosa	Multiple small ulcers (A~S)	Multiple small ulcers or erosions (C~R)	Multiple small erosions (C~S), Bacterial infection	Multiple small erosions (C~D), adenoma (S)	Multiple small ulcers (C~R)	A brownish area in the gastric fornix	
Number of samples obtained (modalities)	n = 1 (surgery)	n = 1 (surgery)	n = 1 (ESD)	n = 1 (biopsy)	n = 5 (biopsy)	n = 5 (biopsy)	N = 5 (biopsy)	n = 5 (biopsy, n = 4; EMR, n = 1)	n = 5 (biopsy)	N = 1 (biopsy)	
Initial histological diagnosis	Lipoma of the right shoulder	Chronic calculous cholecystitis	Early gastric cancer	Chronic gastritis	Ulcers	Ulcers/erosions	Erosions	Ulcers/erosions, adenocarcinoma (Tis, S)	Ulcers	ATTR amyloidosis	
Amyloid deposition (localization)	None	Present (SS)	Present (SM)	None	None	None	Present (C, SM)	Present in the EMR specimen (S, SM)	None	Present (M, SM)	

**Figure 2:**
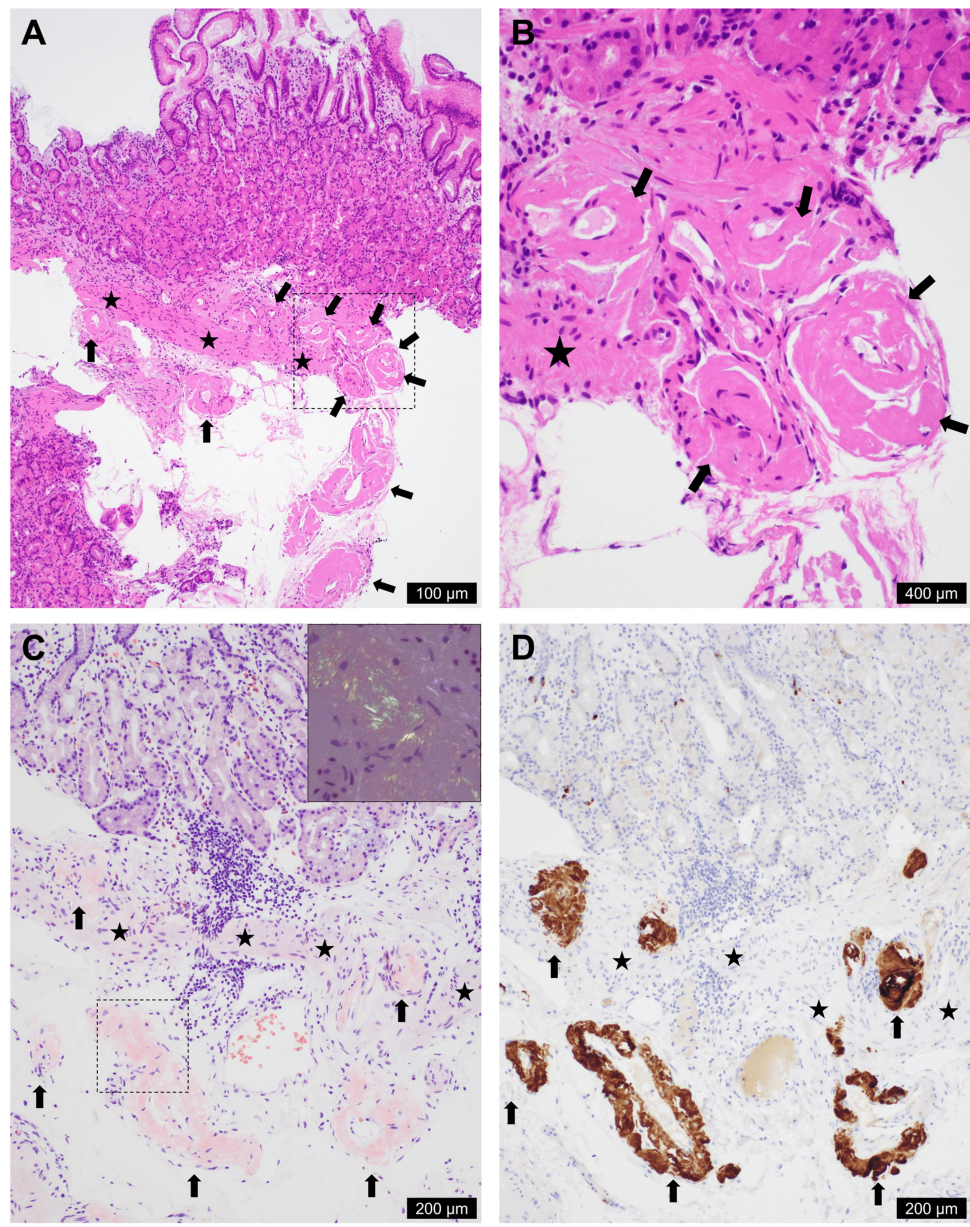
Pathological findings of ATTR amyloidosis on gastric biopsy Hematoxylin and eosin-stained sections of the gastric biopsy (A, B; B corresponding to the dotted square in A) showed mucosal and submucosal blood vessels with thickened walls (indicated by arrows). The thickened vascular walls were stained with Congo red (C, arrows), showing apple-green birefringence under polarized light (C, inset corresponding to the dotted square), and were diffusely and strongly positive for transthyretin (D). Amyloid deposition is indicated by arrows, and the muscularis mucosa is indicated by stars. The scale bars indicate photographs. ATTR: transthyretin amyloidosis

Five colonoscopies, including the first evaluation, were performed during the first year, and each confirmed multiple small ulcers and/or erosions in the entire colon or colorectum. When acute bloody diarrhea occurred, computed tomography confirmed an edematous thick wall of the entire colon and rectum without apparent extravasated contrast materials, and colonoscopy revealed marked mucosal edema and bleeding spots (Figure 1B). The terminal ileum remained intact. A total of 26 colorectal biopsies showed non-specific histological findings, and one colonic endoscopic submucosal resection (EMR) demonstrated intramucosal adenocarcinoma. No vasculitis or cytomegalovirus infection was observed. The bleeding site appeared to be the colorectum; however, the cause could not be determined except for bacterial colitis. Several blood transfusions and iron supplements were used to treat the anemia. The symptoms improved with symptomatic treatment. In the evaluation of the second attack of acute bloody diarrhea, esophagogastroduodenoscopy revealed no bleeding site and an intact duodenal mucosa; however, ATTR amyloidosis was detected in brownish areas of the mucosa in the gastric fornix on biopsy (Figure 1C). A gastric biopsy confirmed mucosal and submucosal thickening of the vessels (Figures 2A, 2B), which were positive for Congo red staining under a microscope (Fig. 2C). Immunohistochemically, the thickened wall was diffusely and strongly positive for transthyretin (Fig. 2D), faintly stained for amyloid A, and negative for kappa light chain, lambda light chain, and beta-2 microglobulin.

**Figure 3:**
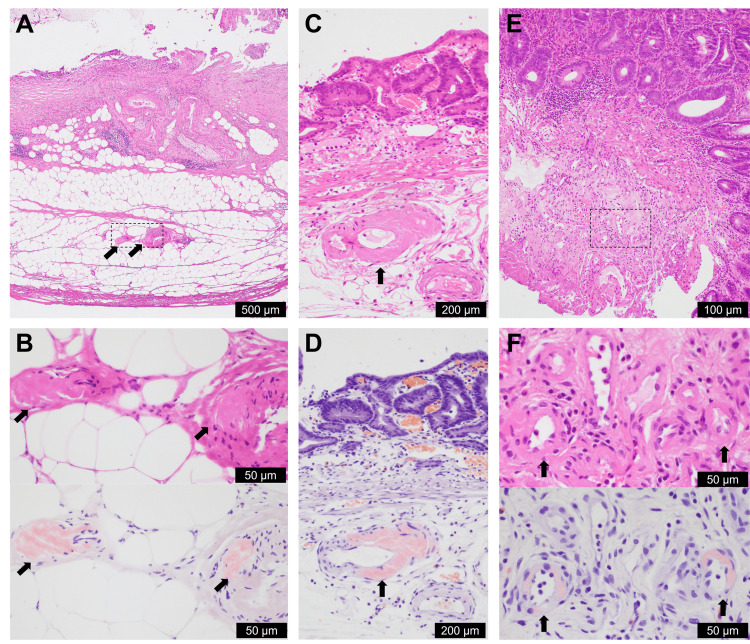
Retrospective pathological findings of ATTR amyloidosis in previously biopsied or resected tissues from the gallbladder, stomach, and sigmoid colon Amyloid deposition, indicated by arrows, was retrospectively detected in the subserosal vessels of the gallbladder (A, B) obtained 12 years earlier for cholecystolithiasis, in the submucosal vessels of the stomach (C, D) obtained by endoscopic submucosal dissection six years earlier for early gastric cancer, and in the submucosal vessels of the sigmoid colon polyp obtained by endoscopic mucosal resection six months earlier (E, F). Amyloid deposition in the sigmoid colon was inconspicuous with hematoxylin and eosin staining in E and F (upper portion). A, B (upper portion), C, E, and F (upper portion): hematoxylin and eosin staining; B (lower portion), D, and F (lower portion): Congo red staining. B and F showed magnified images of the dotted squares in A and E, respectively. The images in B and F are divided into upper and lower sections, but both show almost the same area of the tissue. The scale bars indicate photographs. ATTR: transthyretin amyloidosis

Therefore, a retrospective pathological evaluation for ATTR amyloidosis was performed using the following available tissue samples: shoulder lipoma, gallbladder, gastric ESD, colonic EMR, and the 26 colon biopsies. ATTR amyloidosis was pathologically detected in the gallbladder vascular wall (Figures 3A, 3B), gastric ESD tissue (Figures 3C, 3D), colon EMR tissue (Figures 3E, 3F), and in one of the 26 colon biopsies. Amyloidosis was easily detected in the gastric submucosal vessels.

Clinically, monoclonal proteins were absent in the serum and urine on immunofixation electrophoresis. Echocardiography and electrocardiography revealed no abnormalities. There were no physical findings suggestive of carpal tunnel syndrome or peripheral neuropathy. Dilatation of the digestive tract was inconspicuous on computed tomography. No family history of amyloidosis was noted.

Overall, the anemia appeared to be induced by colonic ulcers and/or erosions caused by ATTR amyloidosis. The patient did not wish to be tested for TTR gene abnormalities. No bleeding events occurred for three months after the second episode of acute bloody diarrhea. The patient provided informed consent for the publication of this report.

## Discussion

Herein, we present a case of ATTR amyloidosis with symptoms and clinical findings of multiple small colorectal ulcers/erosions, positive fecal occult blood tests, and acute bloody diarrhea.

Gastrointestinal manifestations of ATTR amyloidosis are common in ATTRv amyloidosis (varying from 20% to 78%) and rare in ATTRwt amyloidosis (3.8%) [7]. The major GI symptoms of ATTR amyloidosis are early satiety, nausea, vomiting, constipation, diarrhea, and fecal incontinence [6-9]. The differential diagnoses of ATTR amyloidosis with GI symptoms are diverse (infectious colitis, drug-related colitis, irritable bowel syndrome, and inflammatory bowel disease), and ATTR amyloidosis can easily be misdiagnosed [9]. Gastrointestinal bleeding was not reported in 14 patients with gastric ATTR amyloidosis [10]. Colonic bleeding has not been reported as a major GI symptom of ATTR amyloidosis, and acute bloody diarrhea is an unusual symptom of ATTR amyloidosis. Although GI bleeding/ulceration is frequently seen in GI amyloidosis, the main cause is likely to be AL amyloidosis [11, 12]. Ulcers and/or aphthous lesions in the colon or stomach are atypical in patients with ATTR amyloidosis, as the main endoscopic finding of ATTR amyloidosis is a fine granular appearance [9, 10, 13]. ATTR amyloidosis cannot be suspected based on the clinical and colonoscopic findings in this case; however, if multiple persistent ulcers and erosions are observed throughout the colon or colorectum, ATTR amyloidosis should be considered.

The gold standard for a conclusive diagnosis of ATTR amyloidosis involves the histological confirmation of amyloid deposits through biopsy, Congo red staining, and immunohistochemistry. The recommended biopsy sites for ATTRv amyloidosis are the gastrointestinal tract, salivary glands, skin, peripheral nerves, and heart, whereas endomyocardial biopsy is recommended for ATTRwt amyloidosis [2]. ATTR amyloidosis can be detected in the GI tract, from the stomach to the rectum [14]. The esophagus may not be the preferred biopsy site for the pathological diagnosis of ATTR amyloidosis [10]. Since the most common site for detection of TTR amyloid in the GI tract on biopsy is the submucosal vascular wall [14], a biopsy in which the submucosa is sampled is desirable. In addition to the GI tract, a high rate of TTR amyloid detection has been observed in the gallbladder, lungs, and liver [15]. In addition, tissues previously obtained from ligaments associated with carpal tunnel syndrome or lumbar spinal stenosis are useful for diagnosing ATTR amyloidosis.

However, amyloid deposition may not be detected by biopsy. Amyloid deposition in the colon might be pathologically underestimated in our case because only two of the 26 colonic mucosal biopsy tissues contained submucosal tissue. The sensitivity of pathological amyloid evaluation varies according to different biopsy sites and staining techniques [13]. It is now recommended to promptly ask for genetic testing and search for a serum and/or urinary monoclonal protein in parallel with amyloid detection in biopsy samples [8]. Genetic testing is recommended in all cases of suspected ATTR amyloidosis to aid in the diagnosis and to identify whether the disease is hereditary [16]. If the genetic results are positive, the patient promptly receives therapy even if the amyloid is not pathologically detectable, and family members may choose to see a genetic counselor and be tested themselves. Genetic testing for TTR could not be performed because we could not obtain consent from the patient; however, we should make every effort to convince the patient to undergo genetic testing.

## Conclusions

We present a case of an elderly patient with ATTR amyloidosis, persistent colonic ulcers/erosions, and repeated episodes of colonic hemorrhage. Although GI symptoms are unusual in patients with ATTR amyloidosis, ATTR amyloidosis should be listed as a differential diagnosis in elderly patients with anemia and endoscopic evidence of multiple colorectal erosions and/or ulcers. Although many biopsy tissues from colonoscopy were obtained in this case, amyloid deposition was hardly detected in the colorectum, possibly because many specimens did not contain submucosal vessels. Previous biopsies or surgical specimens from patients are useful for retrospective evaluation of amyloidosis. Genetic testing for TTR is also important because even if a biopsy does not prove amyloid, it can make the diagnosis of ATTRv amyloidosis, allowing for early treatment.
